# ‘My words become my hands’: Yoga instructors’ experiences of adapting teleyoga in the SAGE fall prevention trial—A qualitative analysis

**DOI:** 10.1177/20552076231185273

**Published:** 2023-07-06

**Authors:** Heidi Gilchrist, Abby Haynes, Juliana S Oliveira, Catherine Sherrington, Lana Clementson, Janetta Glenn, June Jones, Romina Sesto, Anne Tiedemann

**Affiliations:** 1 The University of Sydney, Sydney Musculoskeletal Health, Sydney, Gadigal Country, NSW, Australia; 2 Institute for Musculoskeletal Health, The University of Sydney and Sydney Local Health District, Sydney, Gadigal Country, NSW, Australia; 3 Flow Collective Yoga, Bulli, Australia; 4 Yoga Connect, Sydney, Australia; 5 Omnibody Yoga and Pilates, Sydney, Australia; 6 Yoga To Go Studio, Petersham, Australia

**Keywords:** Yoga, telehealth, complementary therapies, videoconferencing, experience, physical activity

## Abstract

**Objective:**

This research identifies practical lessons regarding the delivery of teleyoga. Our objectives are to (1) describe challenges and opportunities experienced by yoga instructors when moving the Successful AGEing (SAGE) yoga programme online, and (2) describe how yoga instructors adapted to manage the challenges and leverage opportunities presented by teleyoga.

**Methods:**

This study is a secondary analysis of the data from a previous realist process evaluation of the SAGE yoga trial. The SAGE yoga trial is testing the effect of a yoga-based exercise programme on falls among 700 community-dwelling people aged 60+ years. We draw on focus groups and interviews with four SAGE yoga instructors which we analysed using previously developed programme theories combined with inductive coding and an analytical workshop.

**Results:**

The concerns of the yoga instructors about teleyoga can be characterised into four broad issues: threats to safety, altered interpersonal dynamics, facilitating mind–body connection and difficulties with technology. The SAGE instructors identified eight modifications they used to manage these challenges: a 1:1 participant interview prior to programme commencement, more descriptive verbal instructions, increased focus on interoception, increased attention and support, slower more structured class flow, simplifying poses, adapting the studio environment and IT support.

**Conclusions:**

We have created a typology of strategies for addressing challenges in the delivery of teleyoga for older people. As well as maximising engagement with teleyoga, these manageable strategies could be applied by other instructors to a wide range of telehealth classes, improving the uptake and adherence of beneficial online programmes and services.

## Introduction

### Background

Falls are a serious problem for older people, but regular physical activity focusing on balance and strength can reduce older people's risk of falls and improve their physical function and quality of life.^1–4^ Traditional fall prevention exercise programmes often have poor uptake, adherence and retention,^2,5,6^ and so it is important to find a wide range of activities which engage older people that are beneficial to general health and fall reduction.

Yoga holds great promise in this regard as a mind–body practice that combines strength and balance postures, breathing exercises and concentration,^7^ and which is becoming increasingly popular with middle and older age groups in Australia and internationally.^8–12^ There is good evidence that yoga improves balance and mobility^13^ and health-related quality of life and mental wellbeing for older people,^14^ but as yet there is insufficient evidence to recommend yoga as an evidence based fall prevention strategy.^15,16^

### The Successful AGEing yoga trial

The Successful AGEing (SAGE) yoga trial is a two-arm parallel, pragmatic randomised controlled trial testing the effect of the SAGE yoga-based exercise programme on falls in people aged 60 years and over.^17^ This is the first large (700 participants) randomised controlled trial to test a yoga-based programme designed to prevent falls for people aged 60 and over. Participant recruitment, via advertising in mainstream and social media and community organisations, commenced in October 2019, and the 700th participant was recruited in October 2021. Inclusion criteria were those aged 60 years and over, living independently at home, not currently participating in yoga and able to travel to an intervention location or access online. Participants were randomised to either 12 months of twice-weekly yoga-based exercise delivered by an experienced yoga instructor or two 1-h workshops to learn the seated relaxation yoga programme which they then practised at home, unsupervised for 12 months. Recruitment and enrolment in classes and workshops has occurred monthly on a rolling basis. The yoga-based exercise programme was tested in our pilot trial^18^ and focused on standing yoga postures that build balance and strength, as well as slow breathing and relaxation. The programme was tailored to individual ability and progressed in intensity as participants’ skill and confidence improved: an approach that has been used successfully with older adults.^19^ The primary outcome of interest in the SAGE trial is the rate of falls in the 12-month post-randomisation, and secondary outcomes include mental wellbeing, physical activity, health-related quality of life, balance self-confidence, physical function, pain, goal attainment and sleep quality, and further detail can be found in the protocol.^17^ The SAGE yoga trial received approval from the Human Research Ethics Committee of The University of Sydney (reference 2019/604), and all participants provided written consent. Participants attended an average of 56 classes (range 0–80), and 191 (54%) participants attended at least 80% of the classes on offer. The effect of the intervention on the primary outcome of falls is not yet known as data analysis has not been completed; however, a two-phase process evaluation has been conducted with the first 92 participants to complete the trial and provides the evidence for this paper.

### The transition to teleyoga

Government-mandated lockdowns in Australia during the COVID-19 pandemic, in 2020 and 2021, saw the increased use of telehealth, particularly for vulnerable older populations.^20^ This included the delivery of physical and social interventions for older people to reduce social isolation and decrease functional decline.^21^ Similarly clinical trials of interventions have had to re-think their mode of delivery. Our SAGE yoga trial was no exception.

The SAGE yoga programme was planned to be delivered in live, face-to-face classes in yoga studios; however, due to the pandemic and subsequent social restrictions, programme delivery moved from face-to-face classes to classes provided in real time via interactive videoconferencing, commonly described as teleyoga.^22^

### SAGE yoga trial evaluation of the transition to teleyoga

We conducted a realist evaluation, which is a theory driven approach that aims to explain why a programme works (or doesn’t), for whom and in what circumstances.^23^ This was conducted in parallel with the trial, with the goal of identifying programme theories that link programme activities with information about context, causal mechanisms and outcomes. Programme fidelity is considered to be the extent to which the programme is designed and implemented in a manner congruent with theory.^24^ We have provided a detailed example of this methodological process, using the SAGE trial as an example, elsewhere.^25^ In the Phase 1 evaluation, we identified 16 potential causal mechanisms that could explain outcomes of high satisfaction and adherence among the initial participants (*n* = 43) in the SAGE yoga programme who received a hybrid programme (face-to-face classes for 1–3 months, followed by teleyoga when COVID-19 restrictions came into effect). Phase 2 evaluation considered later recruits (*n* = 49 at the time the evaluation was conducted), who received a wholly online version of the programme.

SAGE trial participants identified several challenges with the online yoga environment. These included the two-dimensional nature of teleyoga and the size of the computer screen (which reduced two-way visibility), communication constraints via video-conferencing (which impacted formal and informal interactions between group members and with instructors) and participants’ home environment (which could be affected by other household members, uncomfortable temperatures and lack of space).^25^ These circumstances have the potential to impact mechanisms of engagement with yoga such as therapeutic alliance, particularly development of a personal bond between the participant and teacher,^26,27^ feelings of connectedness with the class and the instructor^28,29^ and feeling safe and confident of being observed and corrected by the instructor^30,31^ as well as mindfulness^32–34^ and embodiment.^35–37^ Overall, however, participants felt that their yoga instructors were able to address these issues adequately and process data revealed ongoing high rates of satisfaction and adherence across the different modes of programme delivery. Phase 2 evaluation showed that a wholly online mode of delivery retained most of the positive aspects of the hybrid programme and even had some benefits.^38^

Others have also shown teleyoga to be a well-accepted alternative to face-to-face yoga for different groups of people, including older adults.^39–43^ What our evaluation has provided us with, and which is absent from the literature to date, is a discussion of how yoga instructors perceive these challenges and if, and how, they adapt their teaching style for teleyoga in order to maintain these important features of social and therapeutic connectedness, safety and mind–body connection.

### Objectives

This paper identifies practical lessons, drawn from the process evaluation of the SAGE yoga programme, regarding the delivery of teleyoga. Our objectives are to (1) describe challenges and opportunities experienced by the yoga instructors when moving the SAGE yoga programme online and (2) describe how yoga instructors adapted to manage the challenges and leverage opportunities presented by teleyoga. We have created a typology of challenges and solutions to provide practical information for yoga instructors and other health and exercise professionals who want to deliver, or who are already delivering, online classes for older people. We also discuss how yoga instructors activated the mechanisms required for a successful yoga programme when delivering teleyoga.

## Methods

### Process evaluation

Our process evaluation was conducted in two phases and used a realist evaluation methodology to explore what worked for whom in the different programme formats of the SAGE programme (i.e., hybrid and teleyoga). Our methodology and subsequent theory development have been detailed elsewhere^25,44^ and involved data collection across several sources and time points. The process evaluation was led by two experienced (more than 5 years), PhD qualified, female qualitative health researchers (AH and HG). Trial participants and instructors had not previously met the evaluation researchers, who were situated outside the trial team and, while colleagues in the same institute as the trial leaders, were not involved in any other aspects of the trial. Participants and instructors were told that the evaluators were interested in understanding what worked and didn’t work for them regarding the SAGE trial. This paper reports on results from a secondary analysis of this data.

### Data collection

The information presented in this paper is derived from six sources: initial online interviews with all three original SAGE trial yoga instructors were conducted (by AH) at the same time as participant interviews (not reported here) to develop and test hypotheses about the programme that was delivered versus the programme as intended and allowed us to hear how instructors were adapting their practice to suit the context in order to maintain mechanisms of engagement and develop our programme theories: online participant observation of two online classes (conducted by HG) which allowed us to observe these adaptations first hand; an online progress meeting with the SAGE trial research team including chief investigators, yoga instructors and evaluators (AH and HG), where programme theories were formally presented and discussed; a follow-up online focus group (and one interview) with all four SAGE yoga instructors (three original plus one new instructor interviewed three months later, conducted by AH and HG), which was semi-structured and focused on instructors’ experiences of providing online classes and what teaching techniques they found best supported safe, effective, and engaging teleyoga instruction; and a research team review workshop (led by HG) which included the four yoga instructors. Guides for Phase 1 interviews and Phase 2 focus groups and interview can be found in Supplementary Material. Field notes were taken for participant observation and team meetings. Data saturation was not an issue for the yoga instructor interviews and focus groups as all instructors agreed to participate.

Multiple sources of data allowed for analytic triangulation. The SAGE trial yoga instructors are considered part of the research team and are included as co-authors on this paper, so the research team workshop, as well as providing an opportunity for further data collection, served the purpose of member checking (that is ensuring the data truly represented the yoga instructors’ experiences), thus enhancing the trustworthiness of the data.^45^

### Data analysis

Analysis was conducted in parallel with data collection in each phase collaboratively by AH and HG. Yoga instructor interviews in Phase 1 ranged from 32 to 63 min long, and, in Phase 2, the focus group and interview were both ∼60 min long. Interviews and focus groups were audio-recorded and transcribed by a professional transcription company. Digital field notes were taken during the observations. All transcripts and notes were uploaded into NVivo^46^ where they were coded deductively to our existing programme theories and inductively as we identified potential new theories, to develop a framework of realist evaluation concepts and programme theories.^47^ In Phase 2, the yoga instructors’ focus group and interview (both ∼60 min long) were also video recorded, transcribed professionally and uploaded into NVivo. Eight inductive subcodes were introduced to capture new emphases in the data. For example, within the theme ‘gains and losses’, where all details about the transition to teleyoga were coded, we added the codes of ‘difficulties’, ‘benefits’, and ‘impact of moving online’ to the existing codes ‘safety and confidence’ and ‘self-modifications’ (relevant to interviews with participants). Data in the existing ‘suggestions and adaptations’ code were coded more granularly to capture detail in the instructors’ discussions about how they deal with difficulties arising from the online format.

The data collated in these codes were then compared with the programme theories and mechanisms developed in our original evaluation about why the SAGE programme works for most participants,^25^ to see if and how strategies employed by yoga instructors in an online environment support our programme theories and mechanisms.

## Results

### Yoga instructors’ concerns about teleyoga

The four SAGE yoga instructors had significant experience and had been teaching yoga for 12, 15, 25 and 29 years. However, like teachers of all disciplines across the world, the instructors were anxious about moving classes online at the start of the pandemic. While telehealth was not a new concept, these instructors had not taught teleyoga previously, nor had they received any training in online yoga instruction and described feeling overwhelmed:

I was thrown into doing something that I never would, in a million years thought I would be doing when it comes to teaching in the online forum, because it is so **much.** (Instructor 2, Focus Group (FG))

The concerns of the yoga instructors about teleyoga can be characterised into four broad issues: threats to safety, interpersonal dynamics, facilitating mind–body connection and difficulties with technology. We will outline each of these in more detail before going on to describe the modifications instructors used to address these concerns.

#### Safety threats

By far, the largest concern of the instructors was that of the safety of the older people in their classes:

When I was approached to offer the classes online, I had to give this some considerable thought and it was mainly centred on the safety of the participants. How were they going to understand the poses and would they cope? Within a matter of a week, my fears were dispelled, and it was absolutely amazing. (Instructor 1, interview)

The belief that these classes could be delivered safely online was supported by study participants in our earlier research.^25^ However, the instructors quickly learned that it was not a matter of reproducing techniques they used in the studio in front of a camera. Rather, they had to identify difficulties that arose from delivering classes live in the studio, alone and while monitoring and being watched by participants on a computer screen.

The most pronounced difference that our instructors found with teleyoga was the inability to make physical adjustments, which could create the potential for unsafe or suboptimal positioning. In a face-to-face yoga class, as an instructor gives verbal instruction, they will often move around the room and, with permission, use their hands to gently adjust the participant's alignment:

I would say it is very similar to teaching in person. The only difference for me that has been that I can't do any hands on. So it's more of a visual connecting with their energy somehow, for them to do their own adjustments as best they can, without me using touch to help facilitate that. Which I think does get missed, in terms of the nuances, a little bit there. (Instructor 3, FG)

Additionally, the two-dimensional nature of the computer screen created difficulty for the instructors viewing participants’ positions. In the studio, if an instructor cannot see relevant parts of the person's body they will move until they can:

When you see someone on a camera, it depends on the angle the camera's on. Sometimes you’re not even really sure if the foot is really turned out in the direction you want it. There are just certain things that I know now that I can’t see very clearly on every person. (Instructor 2, FG)

This difficulty is particularly evident for instructors while they demonstrate poses:

Another thing that I noticed is the way that you use space to orient yourself. When you’re doing things online, you need to be facing in the one direction at all times and able to see the screen at all times. (Instructor 4, FG)

Equally, the participants had increased difficulty seeing the instructor and each other, and the instructors commented that the online classes initially progressed more slowly than face-to-face classes because of the amount of time they needed to take to get everybody moving together, literally. They describe this as a ‘lag’ for the first month:

… for those first few weeks, it is like almost gathering a herd of cats and kind of bringing them into alignment, so we’re all on the same page. And then the poses can start to flow and the program takes off (Instructor 3, interview).

In the studio, instructors ensure participants safely achieve certain yoga poses by using props (e.g. blocks, straps and cushions) to assist them. In teleyoga, the instructors are relying on participants’ access to materials found in the home to be used as proxy props, which are inconsistent and may be less safe:

We also use a lot of props. So if you can’t touch your toes, I come over and I give you a block and I put that under your hands…But now I’ve got people in different areas around the country in very different environments. Some have walls, some don't have walls. Some have props, some don't have props. You know, even the simple things, like “make sure you have a blanket or a towel”, some towels are fluffy, some towels are not. (Instructor 2, FG)

Lastly, the instructors explained that lack of audio feedback from participants (who are asked to mute) meant they could not hear their breathing, making the intensity of the class more difficult to judge:

When you have someone in front of you [in the studio], you know whether they’re breathing or not. And then that will have a flow-on effect of “how hard it is going to be for them to get out of bed tomorrow?” because they got overworked in that class. (Instructor 2, FG)

#### Altered interpersonal dynamics

The use of teleconferencing platforms (such as Zoom) to deliver yoga creates a class where the instructor can be heard by all, but there is no possibility of private conversations or a quiet aside as there would be in the studio. Students can also speak to the group as a whole, although in SAGE classes (as with many online group classes) once the class begins, the participants were muted to prevent background noise. If a participant wants to speak to the instructor, they must approach their computer and press a button to unmute. This creates a different interpersonal dynamic, and the class may be experienced as less intimate or personal. This is compounded by the loss of any physical touch or connection. However, having the whole group listening to everybody's questions and answers may create a more communal feel to the class: ‘It becomes more of a group experience, for better or for worse, I guess’ (Instructor 4, interview).

These online yoga classes occurred in the context of the COVID-19 pandemic when Australian government restrictions did not allow group exercise and limited social interaction. The instructors all commented on what a ‘life saver’ teleyoga was in these circumstances:

the lockdowns were pretty intense and there was a lot of anxiety stuff for people. And particularly in this age group, a number of them were quite isolated. And so that [teleyoga] was a positive experience. And by the end of our 40 weeks, they'd developed some connections amongst each other, which they may not have done in that group [face-to-face] experience. (Instructor 4, interview)

The instructors were enthusiastic about the different demographic that they now had in their online classes, including people from remote areas, with chronic conditions and with transport problems:

… there's this reach where there's a lot of those people who I would normally not have been able to work with, because they wouldn’t have been able to drive…. So in that scenario, it's like, “Wow, all these people have come together.” And that feels like a big plus … because people do find that it's so integrated into their lives now. And it's just striking. (Instructor 3, FG)

#### Facilitation of mind–body connection

Yoga movements are performed with accompanying breathing exercises. The instructors all described how important it is in the studio to hear people breathing in unison as they move. This helps the participant to be present and focus, but participants are muted in teleyoga so only the instructor can be heard. Some instructors felt the lack of breathing sounds could lead to a class being a more physical and less mindful experience:

There's an energy exchange that happens in-person, particularly when you’ve got a group of people moving and breathing together, and you create an atmosphere or an experience that I think adds to the ability for people to be really mindful and focused in that present moment awareness. (Instructor 4, interview)

In addition, the instructor has little control over the environment in which participants experience an online yoga class. Distractions such as pets, family members and reminders of unfinished domestic tasks were all identified as issues that could also lead to a less mindful experience:

Even just things like distractions or, you’re laying down and you look over there and you’re like, “Oh my God, what's underneath that cupboard?” (Instructor 4, interview)

#### Difficulties with technology

All yoga instructors and participants were on a steep learning curve with the technology required for teleyoga, and often required assistance from the research team. As Instructor 1 said: ‘I don’t have a good relationship with technology…I never thought I would just sort of go with this’. Similarly, they felt some of the participants struggled initially to set up and use their technology successfully:

There was always like two, maybe one, in each group, and they felt so lost and so flustered, they really needed that support. (Instructor 1, FG)

The instructors also commented that if they had not been able to support the participants, particularly in the first few weeks then:

I think we definitely would've lost people to the technology… There were some people that it really was about, “Can I get my head around this technology?” (Instructor 2, FG)

While it is difficult to know how this would be outside of pandemic times, instructors all agreed that there was no going back to face-to-face instruction for many of their participants, since the convenience of the online format outweighed any negatives:

But of the older demographic…the only classes I still have running online (after social restrictions have finished), are my seniors classes. And I have up to 20 people in those, in any given class. I have one (senior) class in the studio, but two classes online (Instructor 2, FG).

### Modifications made by SAGE trial yoga instructors to suit the online format

The yoga instructors all developed techniques for dealing with the differences between the studio and teleyoga classes, often through trial and error. They commented that although it had been a steep learning curve, the process had made them better instructors:

It's made me a better teacher…it's made me have to go back to my basics and revisit the basics. It's forever the student, we’re always learning, but we don’t always keep relearning the foundational work. We have to keep going back there. (Instructor 2, FG)

#### Eight essential techniques

Eight techniques were identified as essential to their revised practice as teleyoga instructors and are described below. They have also been mapped to the challenges they relate to in Table 1.

**Table 1.** A typology of strategies for teleyoga, classified by the four areas of concern raised by the yoga instructors.

Delivering teleyoga – instructors’ concerns	Specific challenge or opportunity	Strategies adopted by yoga teachers
1. Safety threats	Unable to give physical adjustments to correct posture which provides safety and feeling of care and attention Difficulty observing body angles and positions (both instructor and participant) due to two-dimensional screen and home environment Lack of/different props for safe positioning at home Cannot hear participant breathing to judge intensity	Slower, more structured classes, less flow (for this age group), more demonstration Modification of poses and the environment Clearer verbal instruction, for positioning and to encourage personal responsibility 1:1 interview to assist in set up, review props at home, any physical limitations and provide sense of caring
2. Altered interpersonal dynamics between instructors and participants	Unable to have private conversation—lack of more intimate connection Participants less likely to speak up in casual interaction More communal, shared experience Social connection in times of isolation Diverse group of people brought together online	Increased individual attention and support through email, text, after class hours and increased questioning Calling out participants’ names to create connection 1:1 interview to begin to develop rapport
3. Facilitation of mind–body connection	Inability to hear others breathing in unison Distractions in home environment may lead to less mindful practice	Focus on interoception, breathing, internal sensations More breath work
4. Difficulties with technology	Instructor and participants likely to give up if no tech skills	IT support, particularly for first few weeks 1:1 interview to work out any technological issues in advance

##### Introduction of an initial 1:1 interview

When the SAGE trial yoga programme moved online at the start of the pandemic, the instructors advocated for an initial one-on-one interview with participants prior to starting class.

Each participant took part in a private Zoom call with their instructor which lasted ∼15 min. This interview served several purposes. Firstly, it allowed instructors and participants to ‘break the ice’ and develop initial rapport, akin to meeting face-to-face in the studio for the first time:

In a 15-minute time slot, you worked out who the introvert was, who the extrovert was (Instructor 2, FG)

Secondly, it served as an assessment. Participants were asked about their medical history in relation to yoga and if they had any physical issues which concerned them:

I think so much can pass in just having a one-on-one interview of 10, 15 minutes beforehand. Knowing their bodies. Sometimes I would get people to raise their arms up and I’d see where their injury was. And then I was able to kind of really clock that for when we went into open classes. (Instructor 3, FG)

Thirdly, the interview provided an opportunity for the instructors to help participants set up their device and organise the space within their home in which they were going to practise yoga for optimal visibility and safety:

I actually found the interviews…. very good. Because I really used that time, like, “Get your mat. Show me where you're going to put the computer.” And we would spend quite a bit of time, “No, that's not good enough. No, I can’t see you. Come on, let's try it again.” And so we would spend, especially with some people, quite some time just to get the setting right. (Instructor 1, FG)

##### Clearer verbal instructions

All the instructors acknowledged that in the absence of being able to touch or hear a participant, or view them from different angles, their words were even more important than in a face-to-face class:

I find that in the online experience, it requires less silence from the teacher. There's kind of like… you’re filling the gaps, you’re talking through the energy to create the energy verbally, rather than some other means that you might be able to [use] in a group environment in person. (Instructor 4, FG)

However, it was also important not to bombard participants with instructions. One instructor emphasised the need to choose her words carefully and use them sparingly to not overwhelm:

It has taught me…ways I can use my words. So my words are my hands, I find I need to give them lots of time to digest the information. So I don’t talk and talk, I just say a few bullet points and then I just let them be and let them digest that information. (Instructor 1, FG)

This instructor also described the importance of clearly communicating expectations in early classes, so participants appreciate that their safety is paramount:

The other thing is, these initial first few weeks, is also teaching them responsibility for their bodies…. so you’re not having to always sort of look at them and remind them, they need to also remind themselves. So teaching that responsibility, especially for online, I think is a good thing for both parties. (Instructor 1, FG)

Instructions become ‘more global’ and ‘less intricate’. As Instructor 2 said: ‘When I’m teaching online, I am very much barking instructions and it's very broad-brush stroke’.

##### Increased focus on interoception

While proprioception refers to the perception of where one's body, muscles and joints, are in space,^48^ interoception is a technique used in yoga to facilitate mind–body connection by looking inwards and focusing on sensations from inside the body.^49,50^ Instructors described how this was even more important in an online context:

In the absence of having the proprioception of hands-on assistance from the teachers and also in the absence of having as much ability to get that visual aspect of what the teacher's doing…They can see, but being 2D through a screen is a little bit different… I’ve also tried to focus a little bit more on interoception and what the intention of the pose is for them to feel, rather than being so rigid about how it should look in terms of alignment. (Instructor 4, FG)

Similarly, Instructor 3 focused more on the internal experience of breath through Pranayama (breathing exercises) to assist with this connection:

I added in a bit more breathing, a bit of pranayama, bringing that mind-body connection, so they're more grounded in their legs. That was my bridge. (Instructor 3, FG)

##### Calling participants’ names

There was some discussion amongst the instructors about whether to identify participants by name when giving feedback about their posture. Two of the instructors felt it was an important part of the practice to provide individualised instruction. One instructor felt that it helped to sustain connection and hold the participant's attention:

But just by calling their name, I feel like something passes within that online environment. And then also that keeps the thread of me maintaining that real personal connection with them, in terms of zoning in on something within them for that one practise, for that one hour. (Instructor 3, FG)

While the other felt it was important for safety reasons:

When everyone comes on, I go through what my intentions are and I say very clearly that I will be making corrections, especially as this is online content. I'll be coming up very close on occasion to look at everybody…It's simple things, like say, “Gloria, just lift your chest a little bit or notice the difference in your abdominal muscles or your spinal column.” Just little comments like that, where everybody can also go, “Oh, I’m going to lift my chest.” (Instructor 1, FG)

The other two instructors were less inclined to give individualised feedback as they didn’t want individuals to feel criticised but rather gave a general cue or correction to everyone when someone needed to adjust:

I start with the general adjustment …and keep going until they’ve got it. It's a rare occasion that they’re not understanding and then doing it. (Instructor 4, FG)

##### Developing a connection through increased attention and support

However, the above instructor also spoke about how she worked on developing a connection with participants before and after each class:

So we usually talk for a few minutes before class…I just have a little chat with people…It might not necessarily be yoga related. Like for example, a couple of weeks ago…one of the women was putting down her dog that day. So the next week, just checking in and asking how her family's going, and that kind of stuff. (Instructor 4, interview).

All instructors used this technique of a few minutes of ‘small talk’ at the start of class, to varying degrees. Other techniques for showing care and attention were inviting people with questions to stay back after class, making sure the class had their instructor's phone number and email, and regular mail outs:

I also send quite a few emails and just have them on a distribution list, so that they are in the loop …And that constant reminder that they could reach out, because there are some people that just don’t want to tell you what's going on in front of everyone else. (Instructor 2, FG)

Another instructor described how she felt she needed to be a little more ‘on it’ (meaning more attentive to participants’ needs) than in a face-to-face class:

I have to say to them “I noticed you were modifying this pose” …I ask everyone how they’ve been feeling at the start of class and most people just say, “Yeah, fine.” But if I dig deeper, I can find out that there was a little bit more. (Instructor 4, interview)

One instructor emphasised how it was important to, through her attentive words and actions, create a space of safety and care for the participants:

I don’t tell them I’m here to give you a safe space, it's more … I’ll talk to them and say, “So how are you? What are your injuries? Okay, that's fine, we can look after that. Just make sure you don't want to force that…I demonstrate it through the verbal interaction with them, but I’m consciously holding that …they’ve got to feel safe with me, they’ve got to feel cared for. (Instructor 3, FG)

##### Slower, more structured classes

All the instructors described how the type of teleyoga which works best for this age group and level of experience (none of the participants had recently practised yoga) is a structured class with slow, sequential progression and a lot of repetition. This meant that the SAGE programme, with its goal-oriented, predetermined format and slow building of difficulty, was particularly suitable for the teleyoga format:

The SAGE program works so well for online because it is quite structured, the way it comes together, and there isn’t a flow. I mean, I think if you try to link too many things, I don’t think…that worked as well as, “Okay, come back to the mountain pose. Okay, now let's take the legs wide.” (Instructor 3, FG)

The word that comes to mind for me is staccato. Like you do a pose, come out of it, move to another one. So it's easier to teach that online than what I would usually do in a flow class. And I would say, I definitely have to simplify things… You’ve got to strip it back. It can’t be as complex in that online environment. (Instructor 4, interview)

In our research team meeting, Instructor 4 clarified that she is able to teach a more flowing class online successfully but only with more advanced and experienced participants.

Another instructor described how she demonstrated each new exercise three times, from the front, the back and side on, while everyone stopped and watched their screen, so they could see her posture from all angles.

##### Modification of poses and adapting to the environment

As well as a slower pace and pausing between sequences, individualised modifications to poses were considered more necessary working with older people in the online format because of the lack of appropriate props at home as well as the absence of hands-on assistance that would be given in the studio environment.

All of the yoga instructors acknowledged that they did not reach the endpoint of some of the SAGE programme's pose progressions and described postures they had modified so the class could practise them safely:

The reality of it is, when we wrote the program, we were writing a program that we envisaged was going to be in a beautiful big studio with lots of props, with all the space, everything…. then it just got completely stripped back. And, yeah, you just go, “This group's not ready for this.” We haven’t omitted it, but we’ve worked with variations of. So I wouldn’t say that I got that group to the final pose, but I got them as close as I could without putting them in a compromising position. (Instructor 2, FG)

A specific example of this is how one instructor safely modified a single leg standing posture, called half-moon pose, (where the end point is to touch the floor with one hand while standing on one leg):

I have never taken anyone to the floor with half-moon [within SAGE]. We just press our hand onto the chair and they’re successful in being able to take the leg up, either looking down or looking forward…it challenges me as a teacher, how I can get them to do that, but in a safe environment, and with what they can work with [at home]. (Instructor 1, FG)

These modifications were informed by yoga expertise, but instructors also learned to adapt further as they became more experienced in the online format:

In the beginning, it was very much, “Oh, shit, they’ve got different sized chairs and I didn’t take that into consideration, so the pose is going to feel very different for Mary, versus what it does for Paul.” Whereas now, after all this time, I can give them three different scenarios based on the type of chair they’re using. (Instructor 2, FG)

Modifying participants’ position depending on the space they have available to them was also important:

I’ve got a couple of people who just work between their bed and their wardrobe, so there's only one position they can be in. And it's up to me to try and get the best out of them… I’ll just say, “Gloria, just turn sideways. It’ll help me to see you better.” (Instructor 1, FG)

In addition to adapting to the participants’ environment, the instructors acknowledged they needed to set up their own space to optimise visibility for themselves and participants:

One of the first things I did was go out and buy a big screen …because I just couldn’t teach with a laptop. I was like, I can’t have that many people on a screen. (Instructor 2, interview)

The same instructor also commented that she changed the colour of her mat and became more mindful of the colour of clothes she was wearing to improve her visibility.

##### IT support

Throughout the programme and particularly in the first few weeks, SAGE trial research assistants were on hand to guide participants if they had any difficulty logging in and connecting to the class:

I don’t think I could’ve done without it, especially for the first few weeks. In the beginning, I feel like people really needed it…it was very, very, very supportive having them [IT support] there, that we could draw on them. (Instructor 3, FG)

The instructors also benefited from having technology support:

… I appreciate they set up the Zoom link. They set up all the meetings. They emailed it out for everybody. Yeah. They took care of it all. (Instructor 4, interview)

The instructors also acknowledged the determination of participants to pursue the online classes:

My hat goes off to the demographic in particular because I’ve seen it in my regular classes. They were like, “I’m going to give this a go and I’m going to stick with it, and I’m going to persist, even though it's driving me crazy.”…. I still have some people in my current groups that the partner comes along with the iPad every class, he's walking around across the lounge room and he sets everything up, and she's not allowed to touch anything….. So it's not that they’re necessarily any better at technology, but they’re willing to give it a go. (Instructor 2, FG)

## Discussion

This study provides unique insights into the challenges of delivering teleyoga to older people from the perspective of the yoga instructor and identifies the adaptive strategies instructors used to overcome these challenges, presenting them in a useful typology (Table 1). We identified four overarching concerns the yoga instructors had about teleyoga compared to studio-based yoga classes: (a) safety threats—ensuring that participants are practicing yoga safely and *feel* safe while doing so, (b) altered interpersonal dynamics between instructors and participants, (c) barriers to facilitation of mind–body connection and (d) difficulties with technology. We provide examples of the challenges and opportunities which arise from each of these concerns. In a previous evaluation of the SAGE yoga programme, we identified six programme theories configured around 16 mechanisms that explained participants’ engagement in SAGE classes: (1) It's worth the effort; (2) In expert hands, these had the same mechanisms: value expectancy, therapeutic alliance and achievement/mastery; (3) a communal experience (these mechanisms were shared experience, social connection, social comparison and peer checking); (4) putting yoga within reach (accessibility, convenience and gratitude); (5) building yoga habits (purposeful structure, momentum, accountability and continuity); and (6) Yoga's special properties (embodiment and mindfulness).^25^ The yoga instructors’ concerns about online classes touched on all of these but focused on the ways in which online teaching might fail to generate value expectancy (the anticipation of health benefits), therapeutic alliance, embodiment and mindfulness.

### Safety and feeling safe

According to the SAGE trial yoga instructors, in teleyoga, there is potential for participants to adopt unsafe postures for a number of reasons. For example, the small size and two-dimensional nature of the screen, often combined with poor lighting, can make accurate viewing of the participants and instructor more difficult. This means the participant may not feel confident that they have adopted the correct position, and equally the instructor cannot tell if the participant is in the correct position. This problem is compounded for the instructors if the participants are moving at different times, making them harder to observe. In a face-to-face environment where participants can see each other, they tend to move more uniformly as a group, and instructors can walk around the class to view people up close or at different angles, as well as adjust the participant's position if necessary, using their hands. Instructors also use a variety of props in the studio, such as blocks, bolsters, blankets and walls, to aid participants to achieve postures safely. Not being able to hear the participants during a teleyoga class (as they are muted to prevent background noise) also means it is harder to judge how the participants are coping with the class. Some of these issues have been identified in other studies examining teleyoga^39–41,43^ but they have been less specific in how to address them.

Strategies developed by the SAGE trial yoga instructors to ameliorate these safety challenges include providing a more detailed demonstration from more than one angle prior to starting, slowing the pace of the class so everyone is moving together, repeating sequences more often, asking participants to change position so they can be viewed from a different angle and more careful choice of words to give common corrections to all participants but not overload them. Another simple change used by instructors was having as big a screen as possible to maximise their view of participants, as well as considering the colour of their mat and clothing for easier participant viewing. An initial one-to-one interview with participants prior to them commencing class also allowed the instructors to give advice on positioning of the camera for optimum viewing and review props such as chairs and towels that the participant had available to use in the home.

These adaptations, as well as preventing potential harm, help to create a sense of trust and safety in the online environment. This experience of being listened to and adjusted for is known to enhance engagement and adherence in both physical activity and fall prevention programmes for older people face to face.^28,30,31,51^ The yoga instructors identified the importance of transferring this to the online context.

### Instructor/participant connection

The SAGE trial instructors placed great emphasis on developing a connection with each participant. This positive social connection, also known as therapeutic alliance, can enhance the working relationship between the participant and the instructor and supports engagement and adherence in physical activity programmes for older people.^26,52,53^

Given there is less opportunity for interaction with the instructor online (due to being muted during most of the class) and also because of the group nature of any interaction (there are no private verbal conversations), it is essential for instructors to find other ways of facilitating partnership, connectedness and communication in the online environment.^54,55^ The pre-commencement one-to-one interview, as well as providing a good forum to discuss safety and set up, allowed the instructors to learn personal information about the participant privately and kick start a meaningful connection. Instructors also considered each participant's personality type (e.g., introvert or extrovert) to see how they would fit into their class and whether they would be amenable to answering questions in a group environment. Calling out a participant's name when giving feedback in an online class helped participants to feel “seen” and deepened their sense of safety and professional connection. Instructors described having a dedicated time before and/or after class in which participants were invited to ask questions and raise concerns, which also enabled the instructors to ask some probing questions as they would more organically in the face-to-face format. Instructors also sent out emails, encouraged other forms of contact with them (phone and text) and waited after the group class for anyone who might want to stay online and talk to them privately.

The instructors felt that these strategies served a second purpose of creating a sense of community or relatedness among the participants. Social connection formed with others in exercise classes and the sense of a shared experience are supported by self-determination theory and research^29,56,57^ as strong mechanisms for physical activity engagement and adherence among older people.

### Mind–body connection

In yoga, mindfulness is developed through control of breath and mediation techniques which are used to focus on present moment awareness, and this can help to create a sense of embodiment, i.e., a heightened awareness of being in the body.^34,58^ This mind–body connection is a powerful mechanism of engagement in, and enjoyment of, yoga in general^32,35–37,59,60^ and for older people in particular.^61,62^ In the studio, yoga instructors deliberately cultivate an environment with minimal distractions which helps to facilitate this mind–body connection. They have less control over this in the online format as few participants have dedicated time and space completely free from interruptions. In addition, with the muting of participants, there is a loss of the sound of people breathing which, in the studio, creates a collective energy and encourages others to breathe in time. To address this deficit when delivering teleyoga, instructors more regularly reminded people to breathe than they might in a face-to face-class. Instructors also felt that an increased focus on interoception (the processes through which we receive, access and appraise internal bodily signals^49,50^) served to strengthen mindfulness and embodiment in the online environment. Interoceptive processing can be modulated through sustained attention to respiratory signals and bodily sensations such as pressure, movement and temperature, as well as cognitive and affective qualities of sustained focus without judgement.^63^ These techniques have long been used in yoga to enhance mindfulness^64^; however, it is important to use strategies for a more specific focus on this technique in online settings.

### Perseverance with technology

It has been suggested that decreased retention in teleyoga classes for older people is due in part to technological challenges.^43^ The provision of IT support from the research team to both the instructors and the participants in the initial stages of the programme minimised the negative impact of learning new technology and made it easier for participants to persevere, as did having a competent friend or relative that was able to set them up in the initial phases. However, the instructors acknowledged that the participants were willing to persevere with teleyoga even though they found the technology difficult, and this is in keeping with other research demonstrating that older people are willing to persevere through technology difficulties for what they consider high value activities such as exercise.^42^ So provision of technical support is helpful, but the greater value the participants’ place on the class (which is affected by the other mechanisms discussed above), the more likely they will be to continue with teleyoga, despite the difficulties. This is supported by our initial evaluation findings.^25^

### Strengths and weaknesses

To our knowledge this is the first research to describe and collate the experiences of yoga instructors moving from face-to-face to teleyoga for older people, as most research to date has focused on programme feasibility and acceptance from participants’ perspectives.^39–43^ The yoga instructors in the SAGE trial provided practical and useful information about the challenges of teleyoga and how to address these, and their suggestions could be applicable to a range of classes and populations.

While this is only a small sample of four yoga instructors, their experiences were highly similar to each other despite having no regular communication as they made their adaptions. This suggests that the SAGE instructors would have a similar experience to other instructors who moved classes online in response to the pandemic. However, our instructors were able to draw on many years of experience of face-to-face teaching, so less experienced instructors might have a different experience of moving to teleyoga, as might those with previous online teaching experience. Future research could usefully build on these findings by sampling more widely to collate a range of instructors’ experiences, including with different population groups. This would strengthen the confidence with which the strategies suggested here can be extrapolated. We also recognise that the research context of a trial such as SAGE may create different conditions to those experienced by an individual instructor providing community yoga classes, but believe that many of these recommend techniques are likely to be applicable to most circumstances.

## Conclusion

Teleyoga has been shown to be acceptable and feasible for participants, including older people,^39–43^ and increases the reach and cost-effectiveness of yoga for promoting health and wellbeing and reducing risk of falls.^65^ Yoga instructors in the SAGE trial identified four issues with the online environment which could negatively impact engagement and adherence with teleyoga: safety, interpersonal dynamics, mind–body connection and technological confidence.

The SAGE instructors provided eight specific modifications they found useful for tackling these concerns, namely, a 1:1 interview prior to commencement, clearer verbal instructions, increased focus on interoception, increased attention and support, slower more structured class, simplifying poses, adapting their environment and IT support. As well as maximising engagement with teleyoga, these recommendations could easily be applied by other instructors to a wide range of telehealth classes, improving the uptake and adherence of many beneficial online programmes and services.

## Supplemental Material

sj-docx-1-dhj-10.1177_20552076231185273 - Supplemental material for ‘My words become my hands’: Yoga instructors’ experiences of adapting teleyoga in the SAGE fall prevention trial—A qualitative analysis Click here for additional data file. Supplemental material, sj-docx-1-dhj-10.1177_20552076231185273 for ‘My words become my hands’: Yoga instructors’ experiences of adapting teleyoga in the SAGE fall prevention trial—A qualitative analysis by Heidi Gilchrist, Abby Haynes, Juliana S Oliveira, Catherine Sherrington, Lana Clementson, Janetta Glenn, June Jones, Romina Sesto and Anne Tiedemann in DIGITAL HEALTH

sj-docx-2-dhj-10.1177_20552076231185273 - Supplemental material for ‘My words become my hands’: Yoga instructors’ experiences of adapting teleyoga in the SAGE fall prevention trial—A qualitative analysis Click here for additional data file. Supplemental material, sj-docx-2-dhj-10.1177_20552076231185273 for ‘My words become my hands’: Yoga instructors’ experiences of adapting teleyoga in the SAGE fall prevention trial—A qualitative analysis by Heidi Gilchrist, Abby Haynes, Juliana S Oliveira, Catherine Sherrington, Lana Clementson, Janetta Glenn, June Jones, Romina Sesto and Anne Tiedemann in DIGITAL HEALTH

sj-docx-3-dhj-10.1177_20552076231185273 - Supplemental material for ‘My words become my hands’: Yoga instructors’ experiences of adapting teleyoga in the SAGE fall prevention trial—A qualitative analysis Click here for additional data file. Supplemental material, sj-docx-3-dhj-10.1177_20552076231185273 for ‘My words become my hands’: Yoga instructors’ experiences of adapting teleyoga in the SAGE fall prevention trial—A qualitative analysis by Heidi Gilchrist, Abby Haynes, Juliana S Oliveira, Catherine Sherrington, Lana Clementson, Janetta Glenn, June Jones, Romina Sesto and Anne Tiedemann in DIGITAL HEALTH

## References

[1] WHO. WHO guidelines on physical activity and sedentary behaviour: World Health Organization. World Health Organisation (WHO), 2020.

[2] Ng CACM Fairhall N Wallbank G , et al. Exercise for falls prevention in community-dwelling older adults: trial and participant characteristics, interventions and bias in clinical trials from a systematic review. BMJ Open Sport Exerc Med 2019; 5: e000663-e. 10.1136/bmjsem-2019-000663 PMC6936986 31908838

[3] James SL Lucchesi LR Bisignano C , et al. The global burden of falls: global, regional and national estimates of morbidity and mortality from the Global Burden of Disease Study 2017. Inj Prev 2020; 26: i3–i11. 31941758 10.1136/injuryprev-2019-043286 PMC7571347

[4] WHO. *Injuries and violence: the facts* . Report No.: 9241508019. Geneva: WHO Department for the Management of Noncommunicable Diseases, Disability, Violence and Injury Prevention, 2014.

[5] Bunn F Dickinson A Barnett-Page E , et al. A systematic review of older people's perceptions of facilitators and barriers to participation in falls-prevention interventions. Ageing Soc 2008; 28: 449–472.

[6] Robinson L Newton JL Jones D , et al. Self-management and adherence with exercise-based falls prevention programmes: a qualitative study to explore the views and experiences of older people and physiotherapists. Disabil Rehabil 2014; 36: 379–386. 23713970 10.3109/09638288.2013.797507

[7] Tew GA Ward L Hewitt C , et al. Does yoga reduce the risk of falls in older people? Br Med J 2020; 370: m3246. 32883704 10.1136/bmj.m3246

[8] Vergeer I Bennie JA Charity MJ , et al. Participation trends in holistic movement practices: a 10-year comparison of yoga/pilates and t'ai chi/qigong use among a national sample of 195,926 Australians. BMC Complement Altern Med 2017; 17: 296. 28587599 10.1186/s12906-017-1800-6 PMC5461749

[9] Barnes PM Powell-Griner E McFann K , et al. Complementary and alternative medicine use among adults: United States, 2002. Sem Integr Med 2004; 2: 54–71. 15188733

[10] Barnes PM Bloom B Nahin RL . Complementary and alternative medicine use among adults and children: United States, 2007, 2008.

[11] Clarke TC Black LI Stussman BJ , et al. Trends in the use of complementary health approaches among adults: United States, 2002–2012. Natl Health Stat Report 2015; 79: 1. PMC4573565 25671660

[12] Ding D Stamatakis E . Yoga practice in England 1997-2008: prevalence, temporal trends, and correlates of participation. BMC Res Notes 2014; 7: 172. 24661723 10.1186/1756-0500-7-172 PMC3987846

[13] Youkhana S Dean CM Wolff M , et al. Yoga-based exercise improves balance and mobility in people aged 60 and over: a systematic review and meta-analysis. Age Ageing 2016; 45: 21–29. 26707903 10.1093/ageing/afv175

[14] Tulloch A Bombell H Dean C , et al. Yoga-based exercise improves health-related quality of life and mental well-being in older people: a systematic review of randomised controlled trials. Age Ageing 2018; 47: 537–544. 29584813 10.1093/ageing/afy044

[15] Tiedemann A O’Rourke S Sherrington C . Is a yoga-based program with potential to decrease falls perceived to be acceptable to community-dwelling people older than 60. Public Health Res Pract 2018; 28: e28011801. 10.17061/phrp28011801 29925087

[16] Lamb SE Jorstad-Stein EC Hauer K , et al. and Prevention of Falls Network Europe and Outcomes Consensus Group. Development of a common outcome data set for fall injury prevention trials: the Prevention of Falls Network Europe consensus. J Am Geriatr Soc 2005; 53: 1618–1622. 16137297 10.1111/j.1532-5415.2005.53455.x

[17] Oliveira JS Sherrington C Lord S , et al. Yoga-based exercise to prevent falls in community-dwelling people aged 60 years and over: study protocol for the Successful AGEing (SAGE) yoga randomised controlled trial. BMJ Open Sport Exerc Med 2020; 6: e000878. 10.1136/bmjsem-2020-000878 PMC7534729 33033622

[18] Tiedemann A O'Rourke S Sesto R , et al. A 12 week Iyengar yoga program improved balance and mobility in older community-dwelling people: a pilot randomized controlled trial. J Gerontol Med Sci 2013; 68: 1068–1075. 10.1093/gerona/glt087 23825035

[19] Roland KP Jakobi JM Jones GR . Does yoga engender fitness in older adults? A critical review. J Aging Phys Act 2011; 19: 62–79. 21285476 10.1123/japa.19.1.62

[20] Hollander JE Carr BG . Virtually perfect? Telemedicine for COVID-19. N Engl J Med 2020; 382: 1679–1681. 32160451 10.1056/NEJMp2003539

[21] Ho V Merchant RA . The acceptability of digital technology and tele-exercise in the age of COVID-19: cross-sectional study. JMIR Aging 2022; 5: e33165. 35294921 10.2196/33165 PMC9009381

[22] Field T . Yoga research: a narrative review. J Yoga Physiother 2020; 8: 49–71.

[23] Wong G Westhorp G Manzano A , et al. RAMESES II reporting standards for realist evaluations. BMC Med 2016; 14: 1–18. 27342217 10.1186/s12916-016-0643-1 PMC4920991

[24] Adams A Sedalia S McNab S , et al. Lessons learned in using realist evaluation to assess maternal and newborn health programming in rural Bangladesh. Health Policy Plan 2016; 31: 267–275. 26104820 10.1093/heapol/czv053

[25] Haynes A Gilchrist H Oliveira JS , et al. What helps older people persevere with yoga classes? A realist process evaluation of a COVID-19-affected yoga program for fall prevention. BMC Public Health 2022; 22: 463. 10.1186/s12889-022-12818-5 PMC8901433 35255864

[26] Babatunde F MacDermid J MacIntyre N . Characteristics of therapeutic alliance in musculoskeletal physiotherapy and occupational therapy practice: a scoping review of the literature. BMC Health Serv Res 2017; 17: 375. 28558746 10.1186/s12913-017-2311-3 PMC5450083

[27] Moore GF Audrey S Barker M , et al. Process evaluation of complex interventions: Medical Research Council guidance. BMJ 2015; 350: h1258. 25791983 10.1136/bmj.h1258 PMC4366184

[28] Stathi A McKenna J Fox KR . Processes associated with participation and adherence to a 12-month exercise programme for adults aged 70 and older. J Health Psychol 2010; 15: 838–847. 20453043 10.1177/1359105309357090

[29] Deci E Ryan R . Self-determination theory. In: Van Lange P Krugkanski A Higgens T (eds) Handbook of theories of social psychology. 1. London: Sage, 2012, pp.416–437.

[30] Franco MR Tong A Howard K , et al. Older people's perspectives on participation in physical activity: a systematic review and thematic synthesis of qualitative literature. Br J Sports Med 2015; 49: 1268–1276. 25586911 10.1136/bjsports-2014-094015

[31] Sherrington C Lord SR Finch CF . Physical activity interventions to prevent falls among older people: update of the evidence. J Sci Med Sport 2004; 7: 43–51. 15214601 10.1016/s1440-2440(04)80277-9

[32] Wheeler MS Arnkoff DB Glass CR . The neuroscience of mindfulness: how mindfulness alters the brain and facilitates emotion regulation. Mindfulness (N Y) 2017; 8: 1471–1487.

[33] Tang Y-Y Hölzel BK Posner MI . The neuroscience of mindfulness meditation. Nat Rev Neurosci 2015; 16: 213–225. 25783612 10.1038/nrn3916

[34] Gaiswinkler L Unterrainer HF . The relationship between yoga involvement, mindfulness and psychological well-being. Complement Ther Med 2016; 26: 123–127. 27261992 10.1016/j.ctim.2016.03.011

[35] Cox AE Tylka TL . A conceptual model describing mechanisms for how yoga practice may support positive embodiment. Eat Disord 2020; 28: 376–399. 32200707 10.1080/10640266.2020.1740911

[36] Mahlo L Tiggemann M . Yoga and positive body image: a test of the embodiment model. Body Image 2016; 18: 135–142. 27434106 10.1016/j.bodyim.2016.06.008

[37] Piran N Neumark-Sztainer D . Yoga and the experience of embodiment: a discussion of possible links. Eat Disord 2020; 28: 330–348. 31922924 10.1080/10640266.2019.1701350 PMC7347427

[38] Haynes A Gilchrist H Oliveira JS , et al. I wouldn’t have joined if it wasn’t online”: understanding older people’s engagement with teleyoga classes for fall prevention. BMC Complement Med Ther 2022; 22: 283. 36324148 10.1186/s12906-022-03756-1 PMC9628174

[39] Donesky D Selman L McDermott K , et al. Evaluation of the feasibility of a home-based TeleYoga intervention in participants with both chronic obstructive pulmonary disease and heart failure. J Altern Complement Med 2017; 23: 713–721. 28654302 10.1089/acm.2015.0279

[40] Huberty J Eckert R Larkey L , et al. Perceptions of myeloproliferative neoplasm patients participating in an online yoga intervention: a qualitative study. Integr Cancer Ther 2018; 17: 1150–1162. 30352518 10.1177/1534735418808595 PMC6247535

[41] Selman L McDermott K Donesky D , et al. Appropriateness and acceptability of a Tele-Yoga intervention for people with heart failure and chronic obstructive pulmonary disease: qualitative findings from a controlled pilot study. BMC Complement Altern Med 2015; 15: 21. 25887324 10.1186/s12906-015-0540-8 PMC4324792

[42] Gell N Hoffman E Patel K . Technology support challenges and recommendations for adapting an evidence-based exercise program for remote delivery to older adults: exploratory mixed methods study. JMIR Aging 2021; 4: e27645. 34889743 10.2196/27645 PMC8704113

[43] Huang AJ Chesney MA Schembri M , et al. Rapid conversion of a group-based yoga trial for diverse older women to home-based telehealth: lessons learned using zoom to deliver movement-based interventions. J Integr Complement Med 2022; 28: 188–192. 35167358 10.1089/jicm.2021.0268 PMC8867109

[44] Haynes A Gilchrist H Oliveira JS , et al. Using realist evaluation to understand process outcomes in a COVID-19-impacted yoga intervention trial: a worked example. Int J Environ Res Public Health 2021; 18: 9065. 34501654 10.3390/ijerph18179065 PMC8431647

[45] Carlson JA . Avoiding traps in member checking. Qualitative Report 2010; 15: 1102–1113.

[46] NVivo 12.6.0 Plus. QSR International, 2018. https://www.qsrinternational.com/nvivo-qualitative-data-analysis-software/home .

[47] Haynes A Gilchrist H Oliveira JS , et al. Using realist evaluation to understand process outcomes in a COVID-19-impacted yoga intervention trial: a worked example. Int J Environ Res Public Health 2021; 18: 9065. 34501654 10.3390/ijerph18179065 PMC8431647

[48] Ferlinc A Fabiani E Velnar T , et al. The importance and role of proprioception in the elderly: a short review. Mater Sociomed 2019; 31: 219–221. 31762707 10.5455/msm.2019.31.219-221 PMC6853739

[49] Todd J Aspell JE . Mindfulness, interoception, and the body. Brain Sci 2022; 12: 696. 35741582 10.3390/brainsci12060696 PMC9220884

[50] Farb N Daubenmier J Price CJ , et al. Interoception, contemplative practice, and health. Front Psychol 2015; 6: 63. 26106345 10.3389/fpsyg.2015.00763 PMC4460802

[51] Costello E Kafchinski M Vrazel J , et al. Motivators, barriers, and beliefs regarding physical activity in an older adult population. J Geriatr Phys Ther (2001). 2011; 34: 138–147. 10.1519/JPT.0b013e31820e0e71 21937904

[52] Moore AJ Holden MA Foster NE , et al. Therapeutic alliance facilitates adherence to physiotherapy-led exercise and physical activity for older adults with knee pain: a longitudinal qualitative study. J Physiother 2020; 66: 45–53. 31843425 10.1016/j.jphys.2019.11.004

[53] Haynes A Sherrington C Wallbank G , et al. Someone’s got my back”: Older people’s experience of the Coaching for Healthy Ageing program for promoting physical activity and preventing falls. J Aging Phys Act 2020; 29: 296 10.1123/japa.2020-0116 32908018

[54] Mathersul DC Mahoney LA Bayley PJ . Tele-yoga for chronic pain: current status and future directions. Glob Adv Health Med 2018; 7: 2164956118766011. 29637012 10.1177/2164956118766011 PMC5888810

[55] De Baets L Vissers D Timmermans A , et al. Remote Physiotherapy Consultations in the Belgian Primary Health Care Context: Lessons learned during the covid-19 pandemic, https://axxon.be/ckfinder/userfiles/files/Meerjarenplan/Remote%20Physiotherapy%20in%20Belgium.pdf (2021).

[56] Morgan GS Willmott M Ben-Shlomo Y , et al. A life fulfilled: positively influencing physical activity in older adults - a systematic review and meta-ethnography. BMC Public Health 2019; 19: 362. 30940111 10.1186/s12889-019-6624-5 PMC6444855

[57] Murcia JAM Román MLDS Galindo CM , et al. Peers’ influence on exercise enjoyment: a self-determination theory approach. J Sports Sci Med 2008; 7: 23–31. 24150130 PMC3763348

[58] Impett EA Daubenmier JJ Hirschman AL . Minding the body: yoga, embodiment, and well-being. Sex Res Social Policy 2006; 3: 39–48.

[59] Cox AE Ullrich-French S Cole AN , et al. The role of state mindfulness during yoga in predicting self-objectification and reasons for exercise. Psychol Sport Exerc 2016; 22: 321–327.

[60] Khanna S Greeson JM . A narrative review of yoga and mindfulness as complementary therapies for addiction. Complement Ther Med 2013; 21: 244–252. 23642957 10.1016/j.ctim.2013.01.008 PMC3646290

[61] Gilchrist H Haynes A Oliveira JS , et al. The value of mind–body connection in physical activity for older people. J Aging Phys Act 2022; 31: 81–88. 35894992 10.1123/japa.2021-0503

[62] Parra DC Wetherell JL Van Zandt A , et al. A qualitative study of older adults’ perspectives on initiating exercise and mindfulness practice. BMC Geriatr 2019; 19: 354. 31865906 10.1186/s12877-019-1375-9 PMC6927182

[63] Weng HY Feldman JL Leggio L , et al. Interventions and manipulations of interoception. Trends Neurosci 2021; 44: 52–62. 33378657 10.1016/j.tins.2020.09.010 PMC7805576

[64] Gibson J . Mindfulness, interoception, and the body: a contemporary perspective. Front Psychol 2019; 10: 2012. 31572256 10.3389/fpsyg.2019.02012 PMC6753170

[65] Hernani MRA Hernani EV . Online yoga as public health support in the time of COVID-19 PandemicOnline yoga as public health support in the time of COVID-19 pandemic. Int J Prog Sci Technol 2020; 21: 331–337.

