# Supplementary material for: ‘My words become my hands’: Yoga instructors’ experiences of adapting teleyoga in the SAGE fall prevention trial—A qualitative analysis
Source: Digit Health. 2023 Jul 6;9:20552076231185273. doi: 10.1177/20552076231185273 (PMC10331186; doi:10.1177/20552076231185273)
Supplement: sj-docx-2-dhj-10.1177_20552076231185273 - Supplemental material for ‘My words become my hands’: Yoga instructors’ experiences of adapting teleyoga in the SAGE fall prevention trial—A qualitative analysis [file sj-docx-2-dhj-10.1177_20552076231185273.docx]

SAGE process evaluation 2: FOCUS GROUP GUIDE

**Yoga Instructors**

**INTRODUCTION**

Welcome everyone. Thank you so much for giving us your time today. I want to let you know I’m recording this session so we capture the information accurately. Is that OK?

Great, let’s start with some very brief introductions. As you know I’m Heidi Gilchrist, a research associate at the Institute for Musculoskeletal Health. I wasn’t involved in the design or set up of the trial but rather was asked along with Abby to evaluate the program. I’m also a part time physiotherapist and an intermittent practitioner of yoga. If I could get everyone else to briefly introduce yourselves and describe your involvement in the SAGE trial. Thanks everyone. It’s fantastic to have your expert input.

As you know we’re meeting today as part of our evaluation of the SAGE program that you’ve been delivering. We hope that learning more about your experience will help us and other researchers design better programs like this in the future. Today, we’re most interested in gathering as much information as we can about what works and what doesn’t in online yoga instruction. We plan to write a paper that focuses on that topic and we would love you to be co-authors on that if it’s of interest to you. We’ll also share some of the ideas we have about what makes the program work for most people, and get your feedback on them.

You’ve been invited today because of your expertise in delivering this program online, so your opinions are extremely valuable to us. We want to know your views even if they’re different to other people’s. In fact, *especially* if they’re different! We want to know about the whole range of views so that we don’t miss anything important. We’ll move around the group so that everyone has a say. I’m sure I don’t need to ask you to be respectful of each other’s views, but in terms of privacy can we please agree that if you talk about this group with anyone outside, you can tell them what was said but don’t name the person who said it. Does that sound OK? Does anyone have any questions before we kick off?

**Main focus group questions**

1. Let’s start by talking about your overall approach to yoga classes in the SAGE program. Have you approached them with the same philosophy and techniques as you would a ‘normal’ yoga class? What are the main differences?
2. What was it like when you first started online classes (whether for SAGE or previously)? Did you have concerns? What were the main challenges?
3. Has the experience of running online classes changed as time has gone on? What’s been the main learning curve for you?
4. Have you modified your teaching techniques for an online format? What have you done? Have you tried anything that didn’t work very well? What advice would you give to other yoga instructors who are preparing to run classes online?
5. Did you make any adaptations to the SAGE yoga program itself, or perhaps focus on some areas more than others?
6. In our evaluation interviews and focus groups with participants they mentioned three issues relating to online classes that we would love to get your views on:
   - Many participants have told us that they like it when their instructor corrects them specifically, using their name. When we talked about that at the last SAGE meeting you had different views about how appropriate that was as a teaching strategy. What are you views now? [*Prompt: They tell us it makes them feel seen and more confident they are getting the most out of their practice*.] Is it easier to name people online because you can see their names on the screen?
   - Some interviewees felt it was harder to forge a relationship with the instructor (or to reach out to them when they were struggling), but many noted ways their instructors were trying to minimise that problem. What are you currently doing and how effective do you find it?
   - Some suspected that the quality of observation and correction is less effective online: the instructor’s view is 2D, they can’t spot subtle physiological signs, alignment issues or provide hands-on correction. What do you think? Does it potentially make online classes less safe? What strategies are you using to address this?
7. How important was the technology support provided by the SAGE team? How did you use it and what do you think would have happened without it?
8. Is there anything a program like SAGE could do better to help people keep going with yoga after the program finishes?

**Testing program theories**

We have six theories about what makes these yoga classes work for most people. Some of you heard a previous version of these, but they’ve changed quite a bit so we’re keen to revisit them. We’ll show you each of the theories now and get your views on them [share screen with theory slides]. OK, so we think:

1. People enroll in and stick with the program because they anticipate health benefits and then very early on they start to experience health benefits. We think this is just as true of online classes as it is of studio classes.
2. Another reason people value the program, is the quality of yoga instructors: the way they relate to their students, take account of their different needs and support them to achieve what they can for their capabilities. How is this affected by online instruction?
3. Doing yoga live and in a group gives a kind of communal experience which makes the classes feel more meaningful than following a video online. This is less powerful online, but still seems to work.
4. Accessibility and convenience of online classes adds value. This was amplified by COVID-19 restrictions, but maybe people will value online classes less when restrictions lift?
5. The structure of SAGE (twice weekly classes over 40 weeks plus homework resources) helps build yoga habits because it creates a sense of momentum and purpose, and there’s some accountability too. This probably works just as well online as it does in the studio.
6. People engage with yoga differently to other forms of physical activity. We think this is because yoga is holistic, combining physical movement with mindfulness, flow and caring attention to the body. But we’re also getting some feedback that this might be less effective online. What do you think?

**Final thoughts**

1. Is there anything else you can tell us that might help us to develop and improve online yoga programs that aim to support older people to increase strength, flexibility and balance?

***Thank you so much for helping us with our research. It’s been really helpful to hear about your experiences.***
