# Supplementary material for: ‘My words become my hands’: Yoga instructors’ experiences of adapting teleyoga in the SAGE fall prevention trial—A qualitative analysis
Source: Digit Health. 2023 Jul 6;9:20552076231185273. doi: 10.1177/20552076231185273 (PMC10331186; doi:10.1177/20552076231185273)
Supplement: sj-docx-3-dhj-10.1177_20552076231185273 - Supplemental material for ‘My words become my hands’: Yoga instructors’ experiences of adapting teleyoga in the SAGE fall prevention trial—A qualitative analysis [file sj-docx-3-dhj-10.1177_20552076231185273.docx]

**SAGE yoga trial INTERVIEW GUIDE**

Thank you for agreeing to take part in this interview. As you know, we’re evaluating the yoga program that you’ve been taking part in for the past 12 months. We hope that learning more about your experience will help us and other researchers design better programs like this in the future. Our conversation today is confidential which means that when we talk about or publish the results of this evaluation we won’t use any information that reveals your identity. Does that sound OK?

Before we start, I want to let you that I’m **audio recording** our conversation so that I capture your views accurately. Is that OK? Thank you.

Also, can I please double-check that you understand the purpose of this interview and you’re **happy to take part**? Great – thanks!

**Part A. Reasons for participation**

1. Thinking back to when you first heard about the yoga program, what made you decide to take part? What were you hoping to get out of it?
2. What were your views about yoga at the time?
3. What about falls – was that something you had been thinking about?

**Part B. Experiences of participation**

1. We’re keen to learn as much as possible about what worked and what didn’t work for you in the yoga program. I’ll ask you some specific questions about that in a moment. But I wonder if you could start by telling me what your experience of the program was like overall. What was the best thing about it?
2. As you know, due to COVID-19 the yoga classes were changed. You attended face-to-face classes then *took part in online classes/chose to do yoga on your own*. How would you compare the experience of doing yoga in those different ways? What aspects of each work for you? What doesn’t work so well? You said on your feedback form that you preferred __________________________________________________________________________. Can you tell me more about that?

Make sure questions 5-7 are considered for both face-to-face and online classes or self-directed practice: we want to understand the differences

- 1. [if applicable] How do you feel about not being able to attend face-to-face classes due to social restrictions?
  2. [if applicable] What about your connections with peers and the instructor from the group exercise class – has that been affected? Were you able to maintain social connections in the online classes? Do you think it helped that you’d already met face-to-face?

1. We want to understand how a program like this fits in with people’s lives (or, perhaps, doesn’t fit in). Is there anything that gets in the way or makes it difficult for you take part? [If applicable] When we asked you about any barriers to taking part in the program on your feedback form you said:

_____________________________________________________________________________________________________________________ Can you tell me more?

1. Is there anything that helps you stick with the program – makes it easier to keep going?

**Part C. Aspects of the yoga program**

Now I’d like to ask you a few more questions about the detailed feedback you kindly gave us on your feedback form:

1. You scored the yoga instructor ___ out of 10 for how well they delivered the yoga program. Why was that? What was good about her? How could she have made it work better for you?
2. You rated the online yoga classes ______ out of 10. Can you expand on that? What was good about them? What could have made them work better for you?
3. When you were asked *‘Do you have any other comments’* about the program, you said ________________________________________________________________

________________________________________________________________________________________________________________________ Can you expand on that?

**Part D. Impacts and maintenance**

- mental well-being
- PA levels
- attitude to PA
- quality of life
- balance
- self-confidence
- physical function
- pain
- goal attainment
- sleep quality

1. Do you think anything has changed physically or mentally because of taking part in this yoga program?
2. What are your views about yoga now? On your feedback form you rated the program ___ out of 10 for how beneficial you found it. You said:

___________________________________________________________________________________________________________________ Can you tell me more?

1. What about preventing falls - has that changed in any way? Is it an issue for you personally?
2. Thinking about the future now… What do you feel will happen with your yoga practice over the next 12 months? Do you see yourself keeping up with yoga? If not, why? What would help with that?

**Part E. What works? Testing causal hypotheses**

1. We have some theories about what makes these yoga classes work for most people. Can I tell you those theories and get your views on them? OK, so we think:
2. People feel *health benefits* from doing yoga – things like improved balance and mobility which have implications for independence. Has that been your experience? [if yes] But this takes time. What do you think makes people stick with classes early on, before any benefits can be felt? Why did you?
3. The *quality of yoga instructors* is important for making *people feel safe and confident.* And that includes having instructors who understand the needs of older people. What do you think?
4. The *tailoring of the classes* is important too. So our yoga instructors try to adapt exercise according to everyone’s abilities, taking account of their health needs. Is that your experience? Why was that important to you?
5. *Social connections* are an incentive and may add to the *enjoyment* of classes. Was that your experience?
6. Having *no fees* might prompt people to give yoga classes a go and perhaps help them stick with it long-term. Was that a factor for you?

**PART F. Final thoughts**

1. Is there anything else you can tell us that might help us to improve programs like this and to support older people to be as active as possible and avoid falls?

***Thank you so much for helping us with our research. It’s been really helpful to hear about your experience.***
